# Integrative phylogenomics sheds light on the diversity and evolution of fluorescence in coral-dwelling gall crabs

**DOI:** 10.1098/rspb.2024.2403

**Published:** 2025-03-12

**Authors:** Susanne Bähr, Sancia ET van der Meij, Tullia Terraneo, Nicolas Oury, Nico K. Michiels, Stephen Ogg, Fabio Marchese, Francesca Benzoni

**Affiliations:** ^1^ Marine Science Program, Biological and Environmental Science and Engineering Division (BESE), King Abdullah University of Science and Technology, Thuwal, Saudi Arabia; ^2^ Groningen Institute for Evolutionary Life Sciences (GELIFES), University of Groningen, Groningen, The Netherlands; ^3^ Naturalis Biodiversity Center, Leiden, The Netherlands; ^4^ Institute for Evolution and Ecology, University of Tübingen, Tübingen, Germany; ^5^ Imaging and Characterization Core Lab, King Abdullah University of Science and Technology, Thuwal, Saudi Arabia

**Keywords:** adaptive traits, coral-associated decapods, Cryptochiridae, fluorescence evolution, phylogenomics, symbioses

## Abstract

Fluorescence is a notable adaptation in marine environments, helping to counteract the loss of longer wavelengths as light diminishes with depth. Studied to some extent in cnidarians and reef fish, its presence and functions in crustaceans are less understood. Recently, fluorescence was discovered in gall crabs (Cryptochiridae). To investigate the evolutionary significance of fluorescence in these coral-dwelling decapods, we combined a multivariate examination of 27 fluorescent morphological traits with phylogenomic analysis across 14 crab genera from the Red Sea and Indian Ocean. Fluorescence first evolved in the genus *Opecarcinus* and was subsequently retained showing varying levels of expression. We identified four distinct fluorescent morphologies (fluotypes) with high phenotypic variability, some of which show distinct distributions across the phylogeny. Along with differences in the crabs’ microhabitats, these findings suggest that fluorescence may be shaped by selective pressures, such as visibility to potential viewers, and could thus play a role in camouflage, aiding concealment against complex coral reef backgrounds. This study provides a deeper understanding of evolutionary dynamics in cryptochirids and introduces a new workflow, providing guidance for future research on fluorescence in marine invertebrates. Further research into behavioural functions and fluorophore identification are required to explain the observed variability in Cryptochiridae.

## 1. Introduction

Natural fluorescence is an optical phenomenon displayed by numerous terrestrial and aquatic metazoans [1–3]. Fluorescent colouration relies on photoactive molecules called fluorophores, which absorb high-energy, shorter-wavelength light (excitation) and re-emit it at longer wavelengths (emission) [4]. In photic marine habitats where light conditions rapidly change with depth, fluorescence may be particularly useful [5]. The absorption of longer wavelengths (yellow-red) from downwelling sunlight by water gradually narrows the spectrum to blue-green light, with red wavelengths disappearing first below 10 m [6–8]. These conditions favour the use of fluorescence by harnessing the remaining short wavelengths to increase visual contrast [9] or to reintroduce colour at depth [5]. Fluorescence evolved independently in various phylogenetically distant marine taxa, probably as a result of the challenges of inhabiting such a spectrally restricted environment [10].

Fluorescence has been documented in numerous marine vertebrates, including amphioxus [11], sea snakes [12], sharks [9], turtles [13], over 180 species of bony fish [14] as well as various marine invertebrate taxa [15–17]. In fact, fluorescence evolution, diversity and functions are mostly studied in marine invertebrates, particularly cnidarians, where the discovery of the green fluorescent protein (GFP) in the hydrozoan *Aequorea victoria* (Murbach and Shearer, 1902) sparked considerable research interest [18]. GFP orthologues have since been identified in other marine invertebrates, including ctenophores, arthropods and cephalochordates, suggesting that GFP may have been present in the last common ancestor of metazoans [10,19]. Cnidarians, particularly scleractinian corals, exhibit a significant diversity of GFP-like proteins [20]. These fluorescent proteins have been linked to various presumed functions, including photoprotection [21], photoacclimation [22], visual contrast [23], as antioxidants [24], protection against herbivory [25] and attraction of symbiotic dinoflagellates [26]. A recent study also suggests a role in prey attraction in mesophotic habitats [27], underscoring the ecological significance of fluorescence in these sessile organisms.

Despite its widespread occurrence in marine invertebrates, the underlying mechanisms and ecological roles of fluorescence remain mostly understudied in many taxa, including major groups such as crustaceans, which are adapted to a plethora of habitats [28]. Early observations of fluorescence in crustaceans were made in the copepod families Pontellidae Dana, 1852−1853 and Aetideidae Giesbrecht, 1892 [29,30], in which seven GFP-like proteins have been identified. Green fluorescence in these groups may act as mate recognition/attraction signal or as countershading mechanism in the water column [29,31]. In the mantis shrimp *Lysiosquillina glabriuscula* (Lamarck, 1818), yellow fluorescence was recorded on the antennal scales and carapace, where it is thought to play a role in inter- and intraspecific signalling. This finding is supported by the correlation of the fluorescence emission and the spectral sensitivity of stomatopods [32,33]. In decapods, fluorescence has been observed in several species of the genus *Portunus* Weber, 1795 [34], though the study was limited to ethanol-preserved specimens and lacked *in situ* observations. Finally, fluorescence has been observed *in situ* in various decapod species and the stomatopod *Odontodactylus scyllarus* (Linnaeus, 1758). However, these observations were purely descriptive, and the authors could not determine the functional roles of fluorescence [35].

Red fluorescence has been described in coral-dwelling gall crabs (Cryptochiridae Paulson, 1875) [36]. Cryptochirids are highly cryptic obligate symbionts of scleractinian corals, residing in specialized dwellings, pits or galls inside their hosts, which can vary significantly in morphology and in some species completely enclose the crabs [37–39]. The diverse dwelling morphologies may influence light availability and visibility of the crabs, potentially driving the evolution of fluorescence as an adaptive trait. Gall crabs associate with a wide range of coral hosts across multiple families, which might further impact the expression and variation of fluorescence [40]. While cryptochirids lead a secluded lifestyle within their coral hosts, their ‘visiting mate’ system requires males to seek out females that permanently reside in their domiciles [41,42], adding another layer of complexity to the potential selective pressures shaping fluorescence in these crabs.

Here, we investigate the occurrence and variability of fluorescence across Red Sea and Indian Ocean gall crab lineages by applying an integrative phylogenomic approach, including a multivariate morphological trait analysis of cryptochirid fluorescence. Specifically, we aim to test three hypotheses: (i) whether fluorescence is present across all studied gall crab taxa, (ii) if fluorescent taxa form a monophyletic group, and (iii) whether fluorescence, when present, exhibits phenotypic variability across genera. By examining fluorescence patterns across different genera, we aim to identify distinct fluorescent morphologies within the Cryptochiridae and explore their evolutionary implications. Understanding how fluorescence has evolved within distinct ecological niches, shaped by the varied morphologies of the dwellings these crabs inhabit, could offer valuable insights into adaptive traits and ecological interactions within this family of coral-associated decapods. Additionally, the methodology developed here can be applied to explore fluorescence in other reef-dwelling species, broadening our understanding of this phenomenon in understudied marine organisms.

## 2. Material and methods

### (a) Field observations and sample collection

Fluorescence of cryptochirids was investigated *in situ* with fluorescence photography using a Sony a7R IV (Sony, Kōnan, Minato, Tokyo, Japan) equipped with a 90 mm macro lens (Sony FE 90 mm f/2.8 Macro G), a yellow barrier filter (#12 Screw-In Filter, 49YK12, The Tiffen Company, Hauppauge, NY, USA) and blue excitation light sources (Light & Motion, SOLA Nightsea Light, NIGHTSEA, Hatfield, PA, USA). Furthermore, an Olympus OMD EM 1 MARK II (Olympus Corporation, Hachioji, Tokyo, Japan) with a 60 mm macro lens (Olympus M. Zuiko Digital ED 60 mm F/2.8) was used to capture fluorescence under natural lighting conditions by adjusting the white balance across various depths.

A total of 286 gall crab specimens of 14 genera were collected between September 2021 and October 2022 at 14 sites in the central Saudi Arabian Red Sea (*n* = 198), and in February 2022 at 13 sites in the Maldives (*n* = 88), by targeting all known host coral genera in both regions (electronic supplementary material, file S1). Crabs were fixed in 75% ethanol and visually identified to the lowest taxonomic level by the second author. The collected materials are deposited at the King Abdullah University of Science and Technology (KAUST). A detailed description of the sample distribution across different datasets can be found in the electronic supplementary material, file S2.

### (b) Imaging and fluorescent trait analysis

Red Sea specimens (*n* = 162) were imaged for fluorescent trait analysis after a thorough cleaning of the carapaces to remove detritus, coral mucus and algae. Imaging was conducted 1–10 days post-fixation in ethanol to prevent interference from chlorophyll fluorescence caused by algae growing on the crabs. To ensure fluorescence stability, images taken after extended preservation were compared with those of freshly collected specimens. Any crabs showing changes in fluorescence levels resulting in altered patterns of fluorescence were excluded from further analysis.

**Figure 1.**
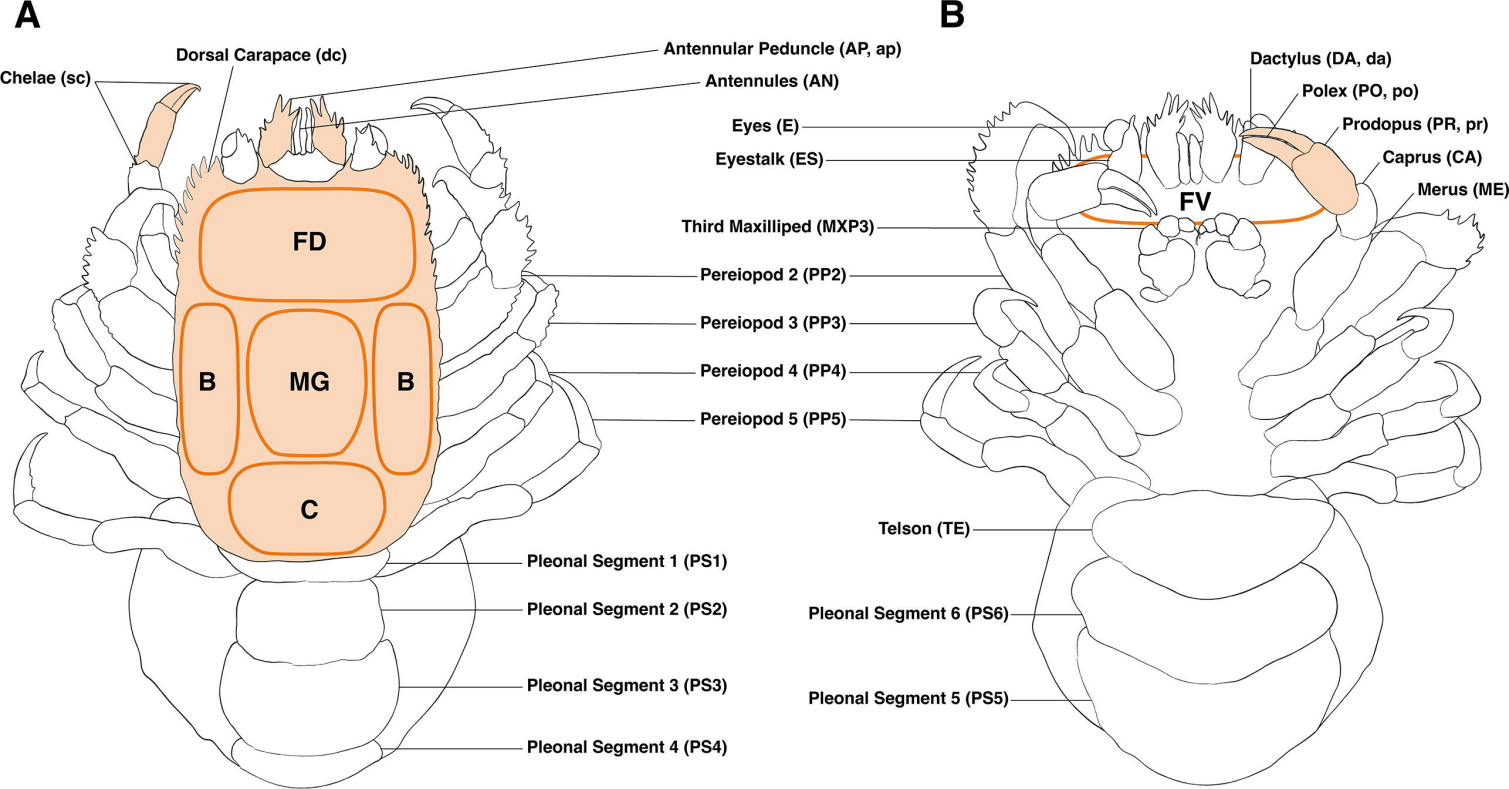
Illustration of a gall crab specimen showing dorsal (A) and ventral (B) views. Fluorescent areas were measured for body parts shaded in orange and indicated by lowercase abbreviations. Body parts analysed for fluorescence morphology are indicated by orange ovals. Capitalized abbreviations indicate body regions examined for the presence or absence of fluorescence. B, branchial; C, cardial; FD, frontal dorsal; FV, frontal ventral; MG, mesogastric.

Images were acquired using a Leica 205A stereomicroscope equipped with a Leica DMC5400 camera and a SFA Stereo Microscope Fluorescence Adapter. The camera was adjusted to ensure each specimen filled the entire field of view, with magnification modified according to specimen size. Images were taken in brightfield and under fluorescence using royal blue (440–460 nm) and green (510–540 nm) excitation lights with corresponding long pass filters. For consistency, exposure time, gain and excitation intensity were adjusted to maximize the dynamic range of the camera. Fluorescence presence or absence was recorded for 25 body parts based on fluorescent light images (figure 1). The dorsal carapace was divided into five regions: frontal dorsal, frontal ventral, mesogastric, cardial and branchial (figure 1). Fluorescent area proportions were quantified for six body parts using grayscale images: dorsal carapace, antennular peduncle, prodopus, polex, dactylus and chelae (figure 1). ImageJ v. 2.3.0 [43] was used for analysis, outlining body parts to first measure their total area and subsequently applying the threshold function to determine their fluorescent area. Finally, the proportion of fluorescent area was calculated by dividing the fluorescent area by the total area. A detailed workflow description and technical specifications are provided in electronic supplementary material, file S3. The Maldives specimens (*n* = 88) were sampled during a liveaboard cruise and could thus only be imaged 12 days post-fixation. Fluorescence presence was confirmed, no further measurements were taken to avoid underestimation and these specimens were excluded from the statistical analysis.

### (c) Spectrometry

Spectrometric measurements to obtain fluorescence emission spectra were collected as described by Meadows *et al*. [44] and Anthes *et al*. [2] using an Ocean Optics (now Ocean Insights) QE65000 fluorescence spectrometer and a bifurcated Ocean Optics QR600-7-UV125BX fibre optics cable. Excitation light was generated using a green laser (ThorLabs CPS532, a 532 nm laser diode module with an AHF narrow-band laser clean-up filter ZET 532/10). The fluorescent signal was maximized by holding the submerged probe at a distance of 4.5−5 mm from the specimen. At this distance, the viewing angle of the central, light-accepting fibre has a diameter of 1.51−1.67 mm (area 1.79−2.19 mm^2^). The fibre guiding the accepted light to the spectrometer included a Semrock EdgeBasic 532 R-25 long-pass filter to eliminate reflected laser light. The spectrometer was calibrated and operated using OceanView 2.0 (Ocean Insights).

### (d) Statistical analyses

The dataset comprised 25 categorical (presence/absence) and 6 continuous (areas) variables describing gall crab fluorescence as well as *sex*, *genus* and *depth* as supplementary variables (figure 1; electronic supplementary material, file S4). A factor analysis of mixed data (FAMD) was performed using the FactoMiner package [45] in R v. 4.2.2 [46]. In this method, quantitative variables are scaled to unit variance, while qualitative variables are transformed into a disjunctive data table and scaled according to multiple correspondence analysis. Proportional data were arcsine transformed, as recommended for data with a high number of zero values [47], and missing values (5.7 %) were imputed using the missMDA package [48,49]. To investigate the similarity of individuals in a multivariate context, we applied Euclidean distance-based methods such as hierarchical and partitional k-means clustering [50]. Within FactoMineR, clustering methods are combined with FAMD through the hierarchical clustering on principal components (HCPC) function. This function applies clustering methods to the first few principal components from the FAMD [50]. Our study applied HCPC to the first six principal components of the FAMD, based on Kaiser’s [51] criterion of retaining components with eigenvalues greater than 1. The function creates a dendrogram and calculates within-cluster inertia. The optimal number of clusters is suggested by identifying where the highest relative increase in inertia occurs.

### (e) DNA extraction and sequencing

For molecular analyses, the fifth pereiopod or, if available, the egg mass was subsampled, and DNA was extracted using the DNEasy® Blood & Tissue kit (Qiagen Inc., Valencia, CA, USA). Extracted DNA quality and quantity were measured using a Nanodrop spectrophotometer (Thermo Fisher Scientific, Waltham, MA, USA) and a Qubit fluorometer (Invitrogen, Waltham, MA, USA) with Qubit dsDNA HS Assay kit (Thermo Fisher Scientific), respectively. Partial COI sequences for identification of the specimens were obtained following the protocol of Bähr *et al*. [37] in collaboration with the KAUST Bioscience Core Lab. Genome-wide sequence data were obtained using an anchored hybrid enrichment (AHE) approach with the Brachyura bait set [52]. AHE library preparation and sequencing in PE150 on an Illumina Novaseq 6000 (Illumina, San Diego, CA, USA) were performed at Arbor Biosciences (Ann Arbor, MI, USA). An overview of both the COI and AHE datasets is provided in the electronic supplementary material, file S2. In the following, the main manuscript focuses on the phylogenomic analysis; therefore, details regarding the methodology (alignment and phylogenetic analyses) and results of the COI dataset are provided in the electronic supplementary material, file S5.

### (f) Sequence assembly and filtering

The AHE dataset included 173 Red Sea and 81 Maldives specimens, with two *Trapezia tigrina* Eydoux & Souleyet, 1842 specimens as outgroups sequenced for this study. Raw reads quality check was performed with FastQC v. 0.11.8 [53] and MultiQC v. 1.9 [54], before and after low-quality base and adapter contamination removal with *cutadapt*, available within Trim Galore! v. 0.6.10 [55]. Subsequently, HybPiper v. 2.17 [56] was used to assemble the loci using the nucleotide sequences of *Eriocheir sinensis* H. Milne Edwards, 1853 [52] as reference throughout the pipeline. Reconstructed loci were then concatenated into a single fasta file. Post-assembly processing was performed following the online documentation of Phyluce [57]. Loci were individually aligned with MAFFT [58] and then subsequently trimmed with Gblocks. Only loci present in at least 50% of the samples were retained and combined into a single partitioned alignment for final analysis.

### (g) Phylogenomic analyses

Phylogenomic analyses were conducted on the high-performance computing cluster at KAUST using maximum likelihood (ML) in IQ-TREE [59]. ModelFinder [60] was used to determine the best-fit nucleotide substitution model based on the Akaike Information Criterion. The AHE tree was built with the TIM2+F+R10 model and 1000 ultrafast bootstrap replicates. Gene and site concordance factors were estimated across the AHE phylogeny.

The evolutionary history of fluorescence was investigated by conducting an ancestral state reconstruction (ASR) on the phylogenomic ML tree using the maximum-parsimony method in Mesquite v. 3.81 [61]. Character states were categorized as absence (0) or presence (1) of fluorescence. To assess the similarity between the phylogenomic and morphology datasets, a Mantel test (vegan package) was applied, testing the correlation between the distance matrices of the dendrogram from the HCPC analysis and the ML phylogenomic tree of the AHE dataset. This test was based on a subset of specimens (*n* = 128), all of which had data available for both the HCPC analyses and the phylogenomic tree.

## 3. Results

### (a) Fluorescent patterns in cryptochirids

**Figure 2.**
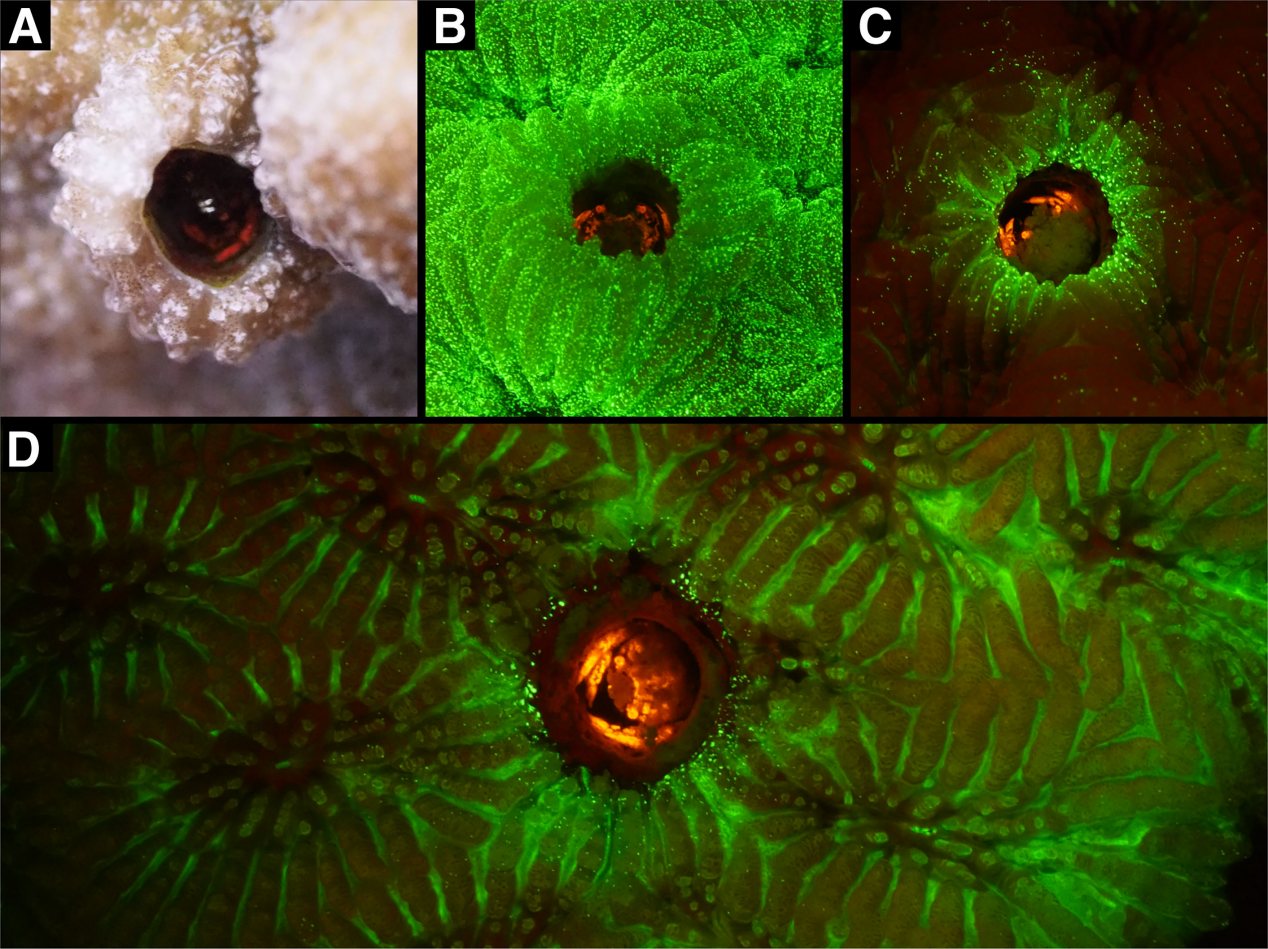
*In situ* observations of fluorescence in coral-dwelling gall crabs. (A) *Lithoscaptus* sp. in *Echinopora* sp. under natural lighting conditions at a depth of over 15 m, where red colours are naturally scarce due to light attenuation. The observed red colouration is attributed to gall crab fluorescence. (B–D) Images captured with royal blue excitation light (440–460 nm) and a yellow barrier filter, revealing the distinct orange fluorescent patterns of *Lithoscaptus* sp. within *Goniastrea pectinata* host colonies.

*In situ*, bright orange/red fluorescence was observed for gall crabs of the genera *Lithoscaptus* A. Milne-Edwards, 1862, *Xynomaia* Kropp, 1990, *Dacryomaia* Kropp, 1990 and *Opecarcinus* Kropp and Manning, 1987 from depths between 7 and 27 m (electronic supplementary material, file S6). In *Lithoscaptus*, fluorescence was successfully captured under natural lighting conditions at depths below 10−15 m (figure 2A). Additionally, fluorescence patterns of this genus were documented across various depths using fluorescence photography (figure 2B–D). For the other cryptochirid genera, this approach failed, linked to their more inconspicuous dwellings along with their reaction to the blue excitation light.

**Figure 3.**
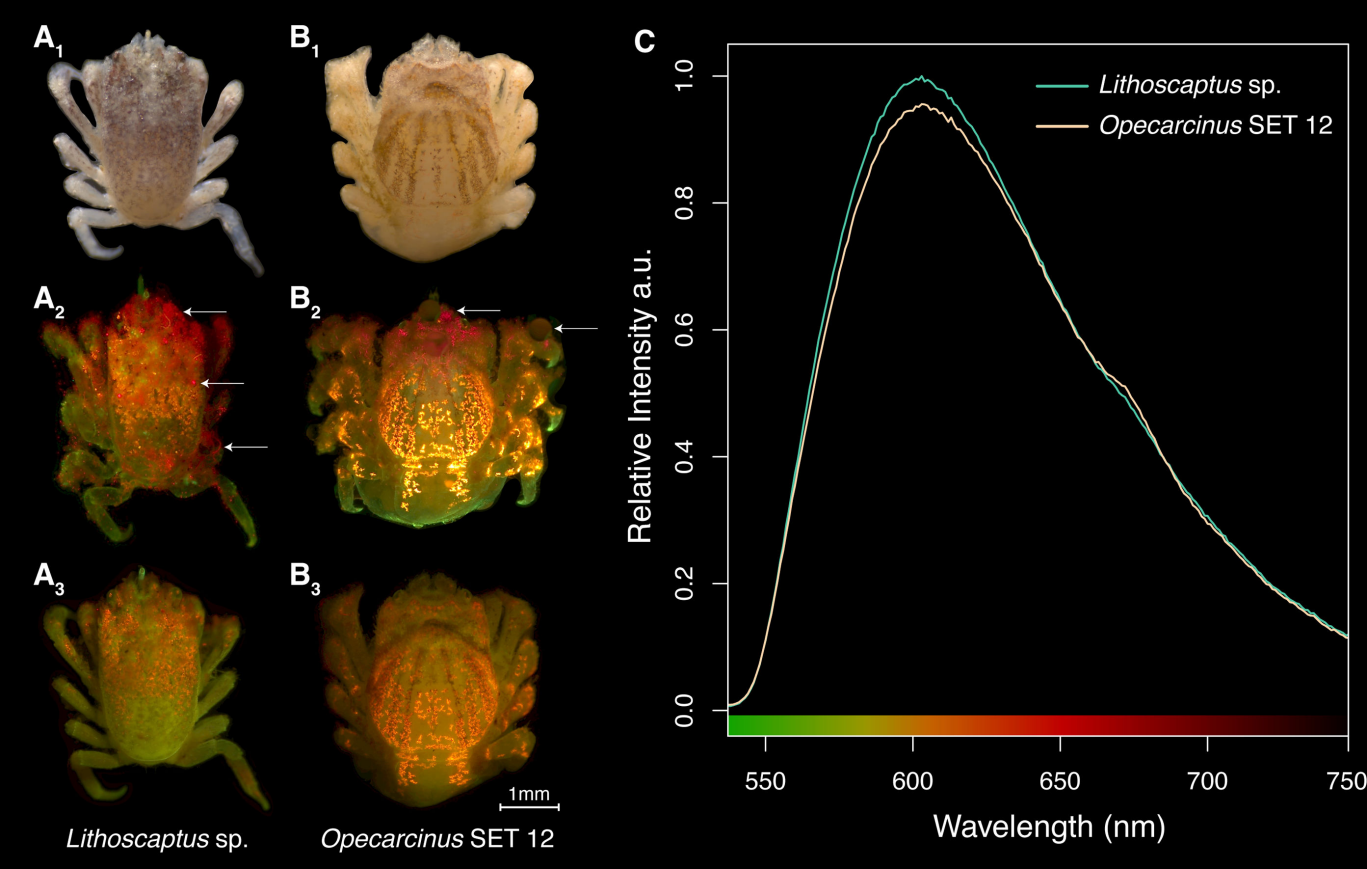
Fluorescence characteristics of two gall crab genera. (A) *Lithoscaptus* sp. specimen under stereomicroscope shown in (i) brightfield setting, (ii) post-extraction appearance under excitation light, and (iii) appearance 9 days post-preservation. (B) *Opecarcinus* SET12 specimen with corresponding imaging conditions to (A). Both genera exhibit green autofluorescence and orange fluorophore fluorescence. White arrows highlight non-fluorescent detritus, algae with red autofluorescence and trapped bubbles—confounding factors in fluorescence signal interpretation. (C) Spectral measurements of red fluorescence in the same Cryptochiridae genera shown in (A and B). Relative intensity of fluorescence emission under monochromatic green excitation light for *Lithoscaptus* (green) and *Opecarcinus* (yellow) is shown.

A total of 250 specimens from the Red Sea and the Maldives belonging to 14 gall crab genera were imaged under the fluorescent stereomicroscope. The tissue and/or exoskeleton of all cryptochirids (pre- and post-fixation in ethanol) exhibited green autofluorescence (figure 3A2−3, B2−3). Additionally, 221 out of the 250 crabs also exhibited fluorescence which appeared as an orange hue when excited by blue light (440–460 nm) and filtered with a 500 nm long pass filter (figure 3A2−3, B2−3). When imaged pre-fixation, specimens were frequently covered in detritus, coral mucus and algae exhibiting chlorophyll fluorescence (figure 3A2–B2), interfering with the orange cryptochirid fluorescence. Post-preservation (1–10) days, confounding effects of chlorophyll fluorescence were eliminated (figure 3A3–B3). Finally, the presence of a single fluorophore with an emission maximum at 603 nm (figure 3C) was confirmed for the two most frequently encountered genera *Opecarcinus* and *Lithoscaptus*.

### (b) Factor analysis of mixed data

A FAMD was conducted on 162 Red Sea gall crab specimens to investigate the phenotypic variability of fluorescence. Initially, the analysis included all 25 fluorescence variables and 6 continuous measures of fluorescent area, with 20.0 % and 13.1 % of the variance explained by the first two PCs, respectively. Body parts consistently lacking fluorescence (e.g. antennules, pleonal segment 6) were subsequently excluded from the analysis, which then explained 33.0 % and 14.6 % of the variance with the first two PCs (electronic supplementary material, file S4). The dorsal carapace, pereiopod 2, dactylus and pleonal segment 3 were strongly determinant in the first two dimensions. The variables dorsal carapace, chelae and dactylus were positively correlated with PC1, indicating their strong association with fluorescence. The categorical variables (figure 1) were split into presence (fluorescence detected) and absence (no fluorescence detected) categories, with absence categories clustering along the negative values of PC1 and presence categories clustering along the positive values (electronic supplementary material, file S4). Genera such as *Neotroglocarcinus* Takeda and Tamura, 1980 and *Pseudohapalocarcinus* Fize and Serène, 1956 were positioned with negative PC1 values, reflecting low fluorescence, whereas *Opecarcinus* and some specimens belonging to *Xynomaia* and *Dacryomaia* showed distinctive fluorescence patterns. Detailed graphs and interpretations are provided in electronic supplementary material, file S4.

### (c) Hierarchical clustering on principal components

**Figure 4.**
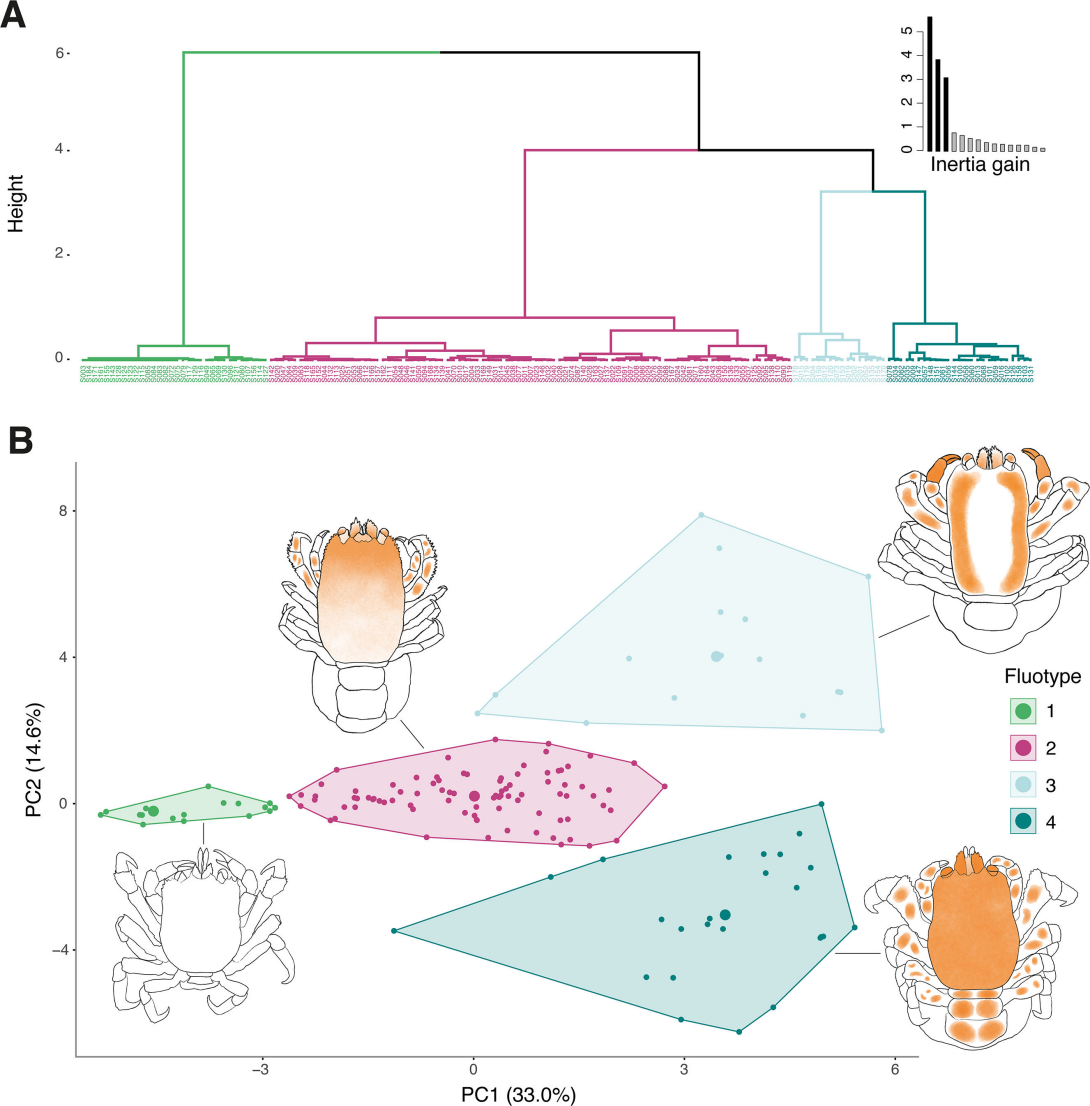
Results of the HCPC analysis. (A) The dendrogram illustrates the hierarchical clustering of specimens based on the first six principal components, with clades colour-coded to represent the four distinct FTs identified. The graph in the upper right corner shows the within-cluster inertia gain, highlighting the optimal number of clusters resulting in the unique FTs. (B) The factor map of the partitional k-means clustering on the first two principal components reveals the separation of specimens into four distinct FTs. Drawings on the plot represent model individuals with representative fluorescence patterns of the FT cluster they are graphically connected to by the line. FT, fluotypes.

The HCPC on the first six PCs of the FAMD (explaining 71.4 % of the total variability) suggested an optimal number of four clusters (figure 4A). The dendrogram revealed one larger cluster including 55 % of the examined specimens and three smaller clusters (figure 4A). This large cluster included specimens with high variability in fluorescence, while the smaller clusters were characterized by specimens with more homogeneous fluorescence patterns. K-means clustering corroborated these findings, with all four clusters being distinct and non-overlapping (figure 4B). The supplementary variable *genus* showed a strong correlation with cluster assignments (χ² = Inf, d.f. = 30, *p* < 0.001), indicating a significant association between crab genera and their fluorescence patterns.

### (d) Fluotype characterization

Based on the significant clustering results, we identified four distinct fluorescent morphologies, which will be referred to as fluotypes (FTs) from this point onward. FT2, the large dendrogram cluster including over half of the specimens, was highly heterogeneous, displaying a wide range of fluorescent patterns across different body parts. In contrast, FT1 contained mostly non-fluorescent specimens, while FT3 and FT4 were characterized by strong fluorescence on specific body parts. FT3 was primarily associated with fluorescence in the chelae, whereas FT4 displayed high fluorescent levels in the pleon and pereiopods. FTs are visually represented in figure 4B where sketches of the FT model individuals highlight the respective fluorescence features. Additional details, including the statistical metrics for each FT, are provided in electronic supplementary material, file S4.

### (e) Phylogenomic analyses

The average number (±s.d.) of raw reads per sample was 3 661 379 (±1 943 228) with a minimum of 5 07 521 and maximum of 19 465 723 reads (see electronic supplementary material, file S5). Sequence data were generated for a total of 490 loci for all 256 samples (including the outgroup specimens; a summary of the trimmed reads, assembled contigs and captured loci are provided in electronic supplementary material, file S5). The number of loci per sample ranged from 3 to 489, with an average of 446 (±40) loci retrieved. Filtering for loci containing sequences for at least 50% of the taxa removed 61 loci, resulting in 429 loci concatenated into an alignment including 217 samples and spanned 90 746 bp. The completeness score for the entire dataset was 0.770. Gaps in the dataset were uniformly distributed with regards to lineage groups. The concatenated alignment contained 32 462 distinct patterns, 30 145 parsimony-informative sites, 6586 singleton sites and 54 015 constant sites. The optimal partitioning scheme generated by IQ-TREE consisted of 38 partitions. The best-fit model for the data was the TIM2+F+R10 model.

**Figure 5.**
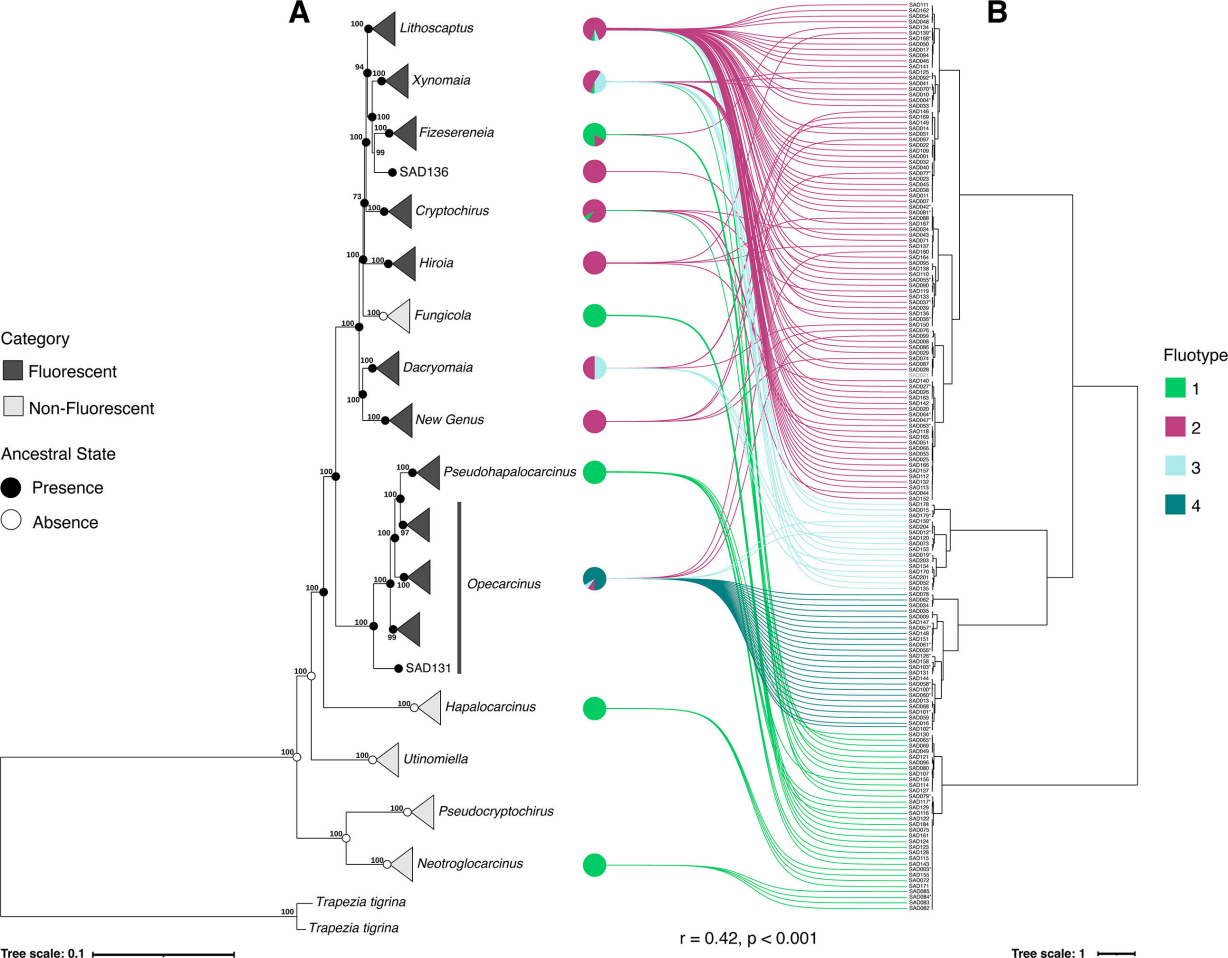
Tanglegram illustrates the relationship between specimen groups obtained by phylogenomic (*A*) and morphological (*B*) analyses of fluorescence in gall crabs. (A) ML phylogenomic tree derived from AHE data, with clades collapsed at the genus level. Nodes include ASR of fluorescence presence (black circles) or absence (white circles). Fluorescent and non-fluorescent lineages are colour-coded (black and light grey, respectively). Bootstrap support values are provided. (B) Dendrogram from the HCPC analysis, with connecting lines linking the dendrogram to pie charts positioned next to each corresponding clade in the phylogenomic tree. Lines are colour-coded based on assigned FT. Results of the Mantel Test are annotated at the bottom (*p*-value based on 999 permutations). Pie charts represent the proportion of specimens within each clade that belong to the four identified FTs. Notably, some specimens represented in the pie charts are absent from the phylogenomic tree, but were identified through a combination of morphological characteristics, host data and COI phylogenetic analysis. Specimens identified but absent from the phylogenomic tree are marked with an asterisk (*) in the dendrogram.

This ML phylogenomic reconstruction resolved 18 highly supported clades (bootstrap values > 98) with *Pseudocryptochirus* Hiro, 1938, *Neotroglocarcinus*, and *Utinomiella* Kropp and Takeda, 1988 recovered as the most basal clades (figure 5A; electronic supplementary material, S5). *Hapalocarcinus* Stimpson, 1859 was inferred as a sister lineage to the remaining clades. *Opecarcinus* specimens were distributed across multiple clades forming three distinct lineages, along with an additional basal single specimen (SAD131) and *Pseudohapalocarcinus,* which was retrieved within this group. *Dacryomaia* was retrieved with a species formerly belonging to *Fungicola,* in a genus that is currently being described and hereafter referred to as ‘new genus’. Together they form a well-supported sister clade to the remainder of the phylogeny. The association of *Fungicola* Serène, 1968 with the remaining clades was strongly supported (bootstrap value = 100), whereas the relationships among the remaining genera displayed the weakest support of the phylogeny (bootstrap value = 73). Within this group, *Fizesereneia* Takeda and Tamura, 1980 was resolved as a well-supported clade with one specimen diverging independently (SAD136). *Lithoscaptus* was retrieved as a monophyletic genus in this phylogeny.

### (f) Evolutionary patterns of cryptochirid fluorescence

To investigate if any relationship could be found between the expression of fluorescence and phylogeny-based lineages in cryptochirids, we annotated the AHE ML phylogenetic reconstruction with fluorescence categories: fluorescent and non-fluorescent. This tree was paired with the morphological dendrogram of the HCPC analysis to visualize possible correlations (figure 5). Our analysis revealed the presence of fluorescence (including minimal signal) across nine genus-level lineages, while five clades were lacking fluorescence based on the examined material (figure 5A). Furthermore, ASR revealed that fluorescence was absent in the common ancestor of cryptochirids and first evolved in the genus *Opecarcinus*. The trait was subsequently retained across lineages, with a single loss observed in *Fungicola*. As stated above, the four FTs (FT1–FT4) identified in the HCPC analysis were tested for correlations with the phylogenetic lineages. The Mantel test of the distance matrices from the dendrogram and AHE phylogenetic tree confirmed a moderate yet significant correlation (*r* = 0.42, *p* < 0.001***, permutations = 999), underscoring the association between genus-level lineages and FTs (figure 5). FT1, mainly characterized by the absence of fluorescence and including only few minimal fluorescent crabs (<10 body parts with spots <50 μm, see electronic supplementary material, file S7), was recurrent throughout the phylogenomic ML tree (figure 5). The highly variable FT2 was strongly associated with *Lithoscaptus*. FT3 was largely confined to *Xynomaia* and *Dacryomaia,* although these genera also contained a notable proportion of FT2 specimens. Finally, FT4 was exclusively found in *Opecarcinus*, underscoring its unique fluorescence profile. Interestingly, the monotypic genus *Pseudohapalocarcinus*, nested within *Opecarcinus*, housed FT1 specimens only.

## 4. Discussion

### (a) Fluorescence in gall crabs

We report orange fluorescence in 9 out of 14 examined gall crab genera from the Red Sea and the Maldives (figure 5*A*). Our sampling covered all but one genus present in the Red Sea (except for *Sphenomaia* Kropp, 1990), contributing significantly to a broader understanding of the prevalence of fluorescence within cryptochirids. Previously, only a few other decapods, such as *Camposcia retusa* (Latreille, 1829), *Scyllarides* sp., *Periclimenes colemani* Bruce, 1975 and *Lybia tessellata* (Latreille in Milbert, 1812), have been documented to exhibit fluorescence [35]. However, most of these observations are limited to a green fluorescent hue recorded *in situ* that might not reflect a visually relevant signal but rather an artefact of their chitinous exoskeletons (e.g. [62]). These findings underscore how little is known about fluorescence in this species-rich order, characterized by highly diversified morphology and a wide range of lifestyles and habitats, from symbiotic to free-living [28].

### (b) Methodological framework

Here, in addition to the widely used COI-based genetic analyses (electronic supplementary material, file S5), we present the first ever phylogenomic reconstruction of the family Cryptochiridae. Currently, the family Cryptochiridae formally comprises 21 genera, 3 of which are restricted to the Atlantic Ocean [63]. Our study included 13 out of the 18 accepted genera recorded across the two regions (i.e. Red Sea and Maldives), with nearly complete representation from the Red Sea (11 out of 12). The AHE approach yielded a well-supported phylogeny (figure 5), underscoring the utility and importance of advanced tools for resolving evolutionary relationships. While the COI and AHE trees were congruent at the clade level, there were some topology differences between the two approaches (electronic supplementary material, file S5). The phylogenomic tree aligns with earlier phylogenetic analyses based on concatenated, multimarker datasets (e.g. [64]), although the position of *Hapalocarcinus* has varied in previous reconstructions. In [64], *Hapalocarcinus* clusters with the three basal-most genera (*Utinomiella*, *Pseudocryptochirus* and *Neotroglocarcinus*), whereas in our tree, it forms a clade with the other derived lineages (figure 5). The placement of *Pseudohapalocarcinus* within *Opecarcinus* is consistent with other studies [65], as is the association of the new genus with *Dacryomaia* and *Fizesereneia* with *Xynomaia* (figure 5) [64]. Notably, we retrieved *Lithoscaptus* as a monophyletic group, whereas it was found to be polyphyletic in other reconstructions (figure 5) [64].

### (c) Fluorescence evolution in gall crabs

Our results indicate that fluorescence first evolved in the common ancestor of *Opecarcinus* and was subsequently retained with variable morphologies across lineages (figure 5A,B). ASR of fluorescence presence/absence suggests a secondary loss of the trait in *Fungicola* (figure 5A). Additionally, certain lineages, such as *Pseudohapalocarcinus* and *Fizesereneia*, exhibit only minimal expression of fluorescence (figure 5; electronic supplementary material, file S7). These findings highlight fluorescence as a trait that, while largely retained across the phylogeny, has undergone reduction or loss in specific lineages. Furthermore, we found fluorescence in cryptochirids to be phenotypically highly variable, which is reflected by different fluorescence levels and patterns across the phylogenetic tree (figures 4 and 5; electronic supplementary material, file S4). Our analysis of fluorescence morphology revealed four distinct FTs (figure 4B). While fluorescence presence/absence could be mapped using ASR, the limited dataset for FTs did not allow us to reconstruct the ancestral traits with this level of detail. Nonetheless, the observed correlation between FTs and the phylogeny provides insights into underlying evolutionary processes influencing fluorescence patterns. In the following sections, we will explore the patterns of fluorescence presence and absence, the distribution of FTs across the phylogeny and their potential evolutionary drivers.

Fluorescence first appears in the highly diverse genus *Opecarcinus*, which typically inhabits open, canopy-like tunnel-shaped dwellings in agariciid coral hosts [38,65]. *Opecarcinus* is also the only genus expressing FT4, characterized by some of the most distinctive fluorescence patterns, including strong fluorescence on the pleonal segments of the abdomen. Viewed in an evolutionary context, these findings imply that when fluorescence first evolved in the family, it involved most body parts, including the abdomen, but was subsequently lost or reduced in other lineages. The drivers for the initial evolution of fluorescence within the Cryptochiridae, and more specifically in the common ancestor of *Opecarcinus*, remain unknown and warrant further investigation. Interestingly, the monotypic genus *Pseudohapalocarcinus*, nested within *Opecarcinus*, exhibits only minimal fluorescence and is exclusively represented by FT1 specimens. The main difference between the two groups is that *Pseudohapalocarcinus* inhabits fully enclosed galls, which suggests that an enclosed habitat may act as a selective pressure against the maintenance of strong fluorescence. Besides variations in fluorescent patterns, it is crucial to recognize the taxonomic complexities within this group: *Pseudohapalocarcinus* is morphologically highly distinct from *Opecarcinus*, and despite co-occurring on the same coral host species as some closely related *Opecarcinus* species, it exhibits entirely different dwelling morphologies [65]. The observed differences in dwelling morphology seem to correlate with fluorescence levels and FT expression in these two genera, suggesting that these factors together contribute to their ecological and evolutionary divergence. Interestingly, *Hapalocarcinus*, another genus residing in enclosed galls [37,66], also lacks fluorescence. However, as it diverged before *Opecarcinus*, the evolutionary reason for the absence of fluorescence remains uncertain.

Beyond the distinct fluorescence expression levels and FT distribution observed in basal clades and *Opecarcinus*, interpreting fluorescence patterns in the remaining phylogeny becomes more challenging due to increased variability and the occurrence of multiple FTs within individual lineages. For instance, in *Fizesereneia*, particularly in *F. panda* [64], fluorescence is generally minimal, with specimens predominantly expressing FT1. This species inhabits large-polyped Lobophylliidae Thiel, 1932 corals with thick fleshy tissue potentially disguising the crabs, reducing the need for fluorescence. However, a single *Fizesereneia* specimen, morphologically similar to *F. panda* but phylogenetically distinct and sampled from an *Acanthastrea* coral H. Milne Edwards and Haime, 1848 with thinner tissue and more conspicuous dwellings exhibited significantly more fluorescence and was characterized as FT2. This suggests that fluorescence may be more advantageous in habitats where the crab’s visibility is less obscured by coral tissue, potentially driving the observed fluorescence variations. Furthermore, this finding hints at potential cryptic speciation within *F. panda*, highlighting the need for further research into fluorescence expression and its ecological drivers in this genus.

In general, FT2 is morphologically highly variable and the most widespread FT across the Cryptochiridae phylogeny, with fluorescent patterns distributed randomly rather than concentrated on specific body parts. It is most prevalent in the large and highly diverse clade of *Lithoscaptus*, a species-rich genus with pervasive and yet unresolved taxonomic issues [67,68]. Furthermore, *Lithoscaptus* species are known to associate with a wide variety of coral hosts belonging to the speciose family Merulinidae H. Milne Edwards and Haime, 1857 [40]. This diversity of host associations, combined with the fact that *Lithoscaptus* inhabits cylindrical pits that do not fully disguise the crabs, could have contributed to the evolution of such a wide array of fluorescence patterns in this genus. The increased visibility of the crabs within their habitats, coupled with ecological interactions driven by host diversity, may have created selective pressures favouring the development of variable fluorescence morphologies. This finding highlights the need for further research into *Lithoscaptus*, including more extensive sampling, detailed examination of fluorescence traits, consideration of coral host associations and efforts to resolve the phylogeny of this complex genus.

The remaining FT3 poses further challenges regarding the interpretation of the observed evolutionary patterns. While observed primarily in *Xynomaia* and *Dacryomaia*, it is also present in other clades indicating the lack of a clear evolutionary signal. Interestingly, this FT is characterized by strong cheliped (prodopus, polex and dactylus) fluorescence. This is particularly intriguing given that chelipeds in other brachyurans are often subject to selective pressures for intraspecific communication. For instance, in *Leptuca leptodactyla* (Rathbun in Ranking, 1898) UV-reflective claws are used in mate recognition [69]. While we cannot conclude its involvement in signalling, its placement on body parts salient to potential viewers, as demonstrated in video evidence (electronic supplementary material, file S8), indicates potential functional significance [32]. However, with limited representation of FT3 in this study, further investigation is needed, particularly with increased sampling of *Dacryomaia* and *Xynomaia*.

While the evolutionary patterns of fluorescence in cryptochirids are complex and not fully resolved, our findings suggest that selective pressures act on both the presence/absence and the expression levels of fluorescence. These pressures appear linked to dwelling morphology, as evidenced by our results, implying that fluorescence likely serves as a visual signal in ecological interactions. However, the case of *Fungicola* and the yet-to-be-described new genus presents a notable exception. Both genera inhabit corals of the family Fungiidae Dana, 1846 with slit-shaped dwelling morphologies, yet *Fungicola syzygia* [64] (to be transferred to the new genus) is fluorescent while *Fungicola* is not [70]. This discrepancy underscores the complexity of fluorescence evolution and suggests that additional factors—potentially genetic, ecological or related to coral host associations—may influence its expression. Further investigations into these genera and their traits could help clarify these unresolved questions. Finally, while our study includes 13 of the 21 formally described cryptochirid genera, broader sampling is essential to fully understand fluorescence evolution in this family. Deep-sea genera, such as *Cecidocarcinus* Kropp and Manning, 1987, and *Zibrovia* Kropp and Manning, 1996, remain unexamined due to the lack of genetic data and recent material. Including these and other missing genera, from both shallow and deep-sea environments, will be critical to capturing the full complexity of fluorescence evolution and its ecological and evolutionary dynamics in cryptochirids.

### (d) Ecological role and visual perception of fluorescence in crustaceans

The general lack of fluorescence records in decapods suggests that the phenomenon is understudied or may not play a significant role in their ecology, especially when compared with reef fish, where fluorescence is more commonly observed and hypothesized to be involved in camouflage, prey detection and communication [2]. For instance, red fluorescence in fish is thought to play an important role in these behaviours due to the specific spectral conditions of the marine environment [5]. In contrast, despite the evolutionary radiation of crustacean eyes, with many taxa possessing well-developed compound eyes [71], their spectral sensitivity is often limited, particularly in decapods, which frequently exhibit monochromatic vision [72]. The spectral sensitivity of gall crabs, in particular, has never been investigated, leaving their ability to perceive their own fluorescence unknown. Interestingly, our field documentation without artificial light sources demonstrated that cryptochirid fluorescence appears as a bright red signal even at depths below 10−15 m, where red wavelengths are absent (figure 2A) [5,8]. This observation suggests that cryptochirid fluorescence, by adding contrast, could function as a perceptible visual signal, akin to those observed in fish. Furthermore, our spectral measurements expand the known wavelength range of crustacean fluorescence from 500 nm in copepods and 524 nm in stomatopods to 603 nm in gall crabs (figure 2C), expanding our understanding of fluorescence in crustaceans.

Considering these observations, it is plausible that fluorescence in gall crabs is subject to selective pressures shaped by their habitats and lifestyle on coral hosts. The variability in fluorescence expression and patterns, combined with differences in dwelling morphologies, supports the hypothesis that fluorescence may serve an ecological purpose, potentially aiding in camouflage. Fluorescence involvement in camouflage has been reported in copepods, where countershading mechanisms obscure visibility in pelagic environments [29]. In reef fish, red fluorescence enhances disruptive colouration, concealing body outlines against complex reef environments and is most commonly observed in benthic species [2]. In cryptochirids, fluorescence patterns, particularly the patchy fluorescence of FT2, may fulfil a similar function, aiding camouflage by disrupting outlines and enhancing concealment against fluorescent coral backgrounds. The distinct localization of fluorescence in FT3 (chelipeds) and FT4 (abdominal fluorescence) remains less understood and warrants further investigation. These FTs may reflect additional ecological functions or selective pressures not yet fully identified. Overall, fluorescence appears to persist as an adaptive trait in cryptochirids, particularly in variable benthic environments like coral reefs, where complex backgrounds and fluctuating light conditions create ecological challenges. Further investigations into the interaction between fluorescence, coral host properties and vision of potential predators, as well as the addition of missing genera, could provide deeper insights into the evolutionary significance of fluorescence in Cryptochiridae.

## 5. Conclusions

Our study reveals that orange fluorescence is both phylogenetically widespread and morphologically diverse in Cryptochiridae, here expressed as four distinct FTs and described for the first time. The distinct distribution of fluorescence and these FTs across the gall crab phylogeny suggests that it is a trait retained with variability across lineages, showing notable shifts in expression levels and high phenotypic variability across the 14 studied genera. Fluorescence in Cryptochiridae may be an adaptive trait influenced by selective pressures, possibly arising from the diversity of coral host associations and the morphology of their dwellings, resulting from subtle differences in the ecological niches they occupy. The variability in fluorescence expression might hint at camouflage as a potential ecological role, though this hypothesis remains speculative. Furthermore, the distinctive placement of fluorescence on chelipeds or abdominal segments in some FTs may indicate additional, unexplored functions. To fully understand the ecological significance of fluorescence in gall crabs, future research should focus on their spectral sensitivity, the visibility of their fluorescence against their often fluorescent coral host backgrounds and the identification of the fluorophore involved. Understanding the biological interactions between cryptochirids and their coral hosts could further illuminate the origins and functions of this trait, especially considering the prevalence and diversity of fluorescence in scleractinian corals. Finally, the widespread presence of fluorescence in other non-fish and coral taxa suggests it may play an underexplored ecological role for these organisms. Given its prevalence, it is plausible that fluorescence serves a purpose beyond what is currently understood, warranting further research into the potential roles of fluorescence in the marine realm. In this context, our newly developed methodology provides an important framework for extending research to additional invertebrate taxa, shedding light on its evolutionary significance across diverse marine environments.

## Data Availability

ESM is available on FigShare [73]. COI sequences have been submitted to GenBank and can be found under the accession numbers PQ373355–PQ373555. AHE sequencing data have been submitted to NCBI Sequencing Read Archive and is available with the BioProject ID PRJNA1168291. Supplementary material is available online [74].

## References

[1] Lagorio M, Cordon G, Iriel A. 2015 Reviewing the relevance of fluorescence in biological systems. Photochem. Photobiol. Sci. **14**, 1538–1559. (10.1039/C5PP00122F) 26103563

[2] Anthes N, Theobald J, Gerlach T, Meadows MG, Michiels NK. 2016 Diversity and ecological correlates of red fluorescence in marine fishes. Front. Ecol. Evol. **4**, 126. (10.3389/fevo.2016.00126)

[3] Olson ER *et al*. 2021 Vivid biofluorescence discovered in the nocturnal springhare (Pedetidae). Sci. Rep. **11**, 4125. (10.1038/s41598-021-83588-0) 33603032 PMC7892538

[4] Johnsen S. 2012 The optics of life: a biologist’s guide to light in nature. Princeton, NJ: Princeton University Press. (10.1063/PT.3.1521)

[5] Michiels NK *et al*. 2008 Red fluorescence in reef fish: a novel signalling mechanism? BMC Ecol. **8**, 16. (10.1186/1472-6785-8-16) 18796150 PMC2567963

[6] Kirk JT. 1994 Light and photosynthesis in aquatic ecosystems. Cambridge, UK: Cambridge University Press. (10.1017/CBO9780511623370)

[7] Loew E, Zhang H. 2006 Propagation of visual signals in the aquatic environment: an interactive windows-based model. In Communication in fishes (eds F Ladich, P Moller, B Kapoor), pp. 281–302. Enfield, NH: Science Publishers.

[8] Jerlov NG. 1968 Optical oceanography. New York, NY: Elsevier.

[9] Gruber DF, Kao HT, Janoschka S, Tsai J, Pieribone VA. 2008 Patterns of fluorescent protein expression in scleractinian corals. Biol. Bull. **215**, 143–154. (10.2307/25470695) 18840775

[10] Macel M, Ristoratore F, Locascio A, Spagnuolo A, Sordino P, D’Aniello S. 2020 Sea as a colour palette: the ecology and evolution of fluorescence. Zool. Lett. **6**, 1–11. (10.1186/s40851-020-00161-9) PMC7288533 32537244

[11] Deheyn DD, Kubokawa K, McCarthy JK, Murakami A, Porrachia M, Rouse GW, Holland ND. 2007 Endogenous green fluorescent protein (GFP) in amphioxus. Biol. Bull. **213**, 95–100. (10.2307/25066625) 17928516

[12] Seiko T, Terai Y. 2019 Fluorescence emission in a marine snake. Galaxea J. Coral Reef Stud. **21**, 7–8. (10.3755/galaxea.21.1_7)

[13] Gruber DF, Sparks JS. 2015 First observation of fluorescence in marine turtles. Am. Mus. Novit. **3845**, 1–8. (10.1206/3845.1)

[14] Sparks JS, Schelly RC, Smith WL, Davis MP, Tchernov D, Pieribone VA, Gruber DF. 2014 The covert world of fish biofluorescence: a phylogenetically widespread and phenotypically variable phenomenon. PLoS One **9**, e83259. (10.1371/journal.pone.0083259) 24421880 PMC3885428

[15] Mehr S, Verdes A, DeSalle R, Sparks J, Pieribone V, Gruber D. 2015 Transcriptome sequencing and annotation of the polychaete Hermodice carunculata (Annelida, Amphinomidae). BMC Genom. **16**, 1–13. (10.1186/s12864-015-1565-6) PMC4462082 26059236

[16] Betti F, Bavestrello G, Cattaneo-Vietti R. 2021 Preliminary evidence of fluorescence in Mediterranean heterobranchs. J. Molluscan Stud. **87**, a040. (10.1093/mollus/eyaa040)

[17] Juhasz-Dora T, James P, Evensen T, Lindberg SK. 2024 Hidden in plain sight: hyperspectral documentation of complex biofluorescence produced by the green sea urchin (Strongylocentrotus droebachiensis). Methods Appl. Fluoresc. **12**, 065008. (10.1088/2050-6120/ad232e) 38277704

[18] Shimomura O, Johnson FH, Saiga Y. 1962 Extraction, purification and properties of aequorin, a bioluminescent protein from the luminous hydromedusan, Aequorea. J. Cell. Comp. Physiol. **59**, 223–239. (10.1002/jcp.1030590302) 13911999

[19] Francis WR, Christianson LM, Powers ML, Haddock SHD. 2016 Non-excitable fluorescent protein orthologs found in ctenophores. BMC Evol. Biol. **16**, 167. (10.1186/s12862-016-0738-5) 27557948 PMC4997694

[20] Alieva NO *et al*. 2008 Diversity and evolution of coral fluorescent proteins. PLoS One **3**, e2680. (10.1371/journal.pone.0002680) 18648549 PMC2481297

[21] Salih A, Larkum A, Cox G, Kühl M, Hoegh-Guldberg O. 2000 Fluorescent pigments in corals are photoprotective. Nature **408**, 850–853. (10.1038/35048564) 11130722

[22] Roth M, Latz M, Goericke R, Deheyn D. 2010 Green fluorescent protein regulation in the coral Acropora yongei during photoacclimation. J. Exp. Biol. **213**, 3644–3655. (10.1242/jeb.040881) 20952612

[23] Gruber D, Kao H, Janoschka S, Tsai J, Pieribone V. 2008 Patterns of fluorescent protein expression in scleractinian corals. Biol. Bull. **215**, 143–154. (10.2307/25470695) 18840775

[24] Palmer CV, Modi CK, Mydlarz LD. 2009 Coral fluorescent proteins as antioxidants. PLoS One **4**, e7298. (10.1371/journal.pone.0007298) 19806218 PMC2752795

[25] Matz MV, Marshall NJ, Vorobyev M. 2006 Are corals colourful? Photochem. Photobiol. **82**, 345–350. (10.1562/2005-08-18-RA-653) 16613484

[26] Aihara Y, Maruyama S, Baird AH, Iguchi A, Takahashi S, Minagawa J. 2019 Green fluorescence from cnidarian hosts attracts symbiotic algae. Proc. Natl. Acad. Sci. USA **116**, 2118–2123. (10.1073/pnas.1812257116) 30670646 PMC6369807

[27] Ben-Zvi O, Lindemann Y, Eyal G, Loya Y. 2022 Coral fluorescence: a prey-lure in deep habitats. Commun. Biol. **5**, 2. (10.1038/s42003-022-03460-3) 35654953 PMC9163160

[28] De Grave S *et al*. 2023 Benchmarking global biodiversity of decapod crustaceans (Crustacea: Decapoda). J. Crustac. Biol. **43**. (10.1093/jcbiol/ruad042)

[29] Shagin D *et al*. 2004 GFP-like proteins as ubiquitous metazoan superfamily: evolution of functional features and structural complexity. Mol. Biol. Evol. **21**, 841–850. (10.1093/molbev/msh079) 14963095

[30] Masuda H, Takenaka Y, Yamaguchi A, Nishikawa S, Mizuno H. 2006 A novel yellowish-green fluorescent protein from the marine copepod, Chiridius poppei, and its use as a reporter protein in HeLa cells. Gene **372**, 18–25. (10.1016/j.gene.2005.11.031) 16481130

[31] Hunt M, Scherrer M, Ferrari F, Matz M. 2010 Very bright green fluorescent proteins from the Pontellid copepod Pontella mimocerami. PLoS One **5**, e11517. (10.1371/journal.pone.0011517) 20644720 PMC2904364

[32] Marshall J, Johnsen S. 2017 Fluorescence as a means of colour signal enhancement. Phil. Trans. R. Soc. B **372**, 20160335. (10.1098/rstb.2016.0335) 28533452 PMC5444056

[33] Mazel CH, Cronin TW, Caldwell RL, Marshall NJ. 2004 Fluorescent enhancement of signaling in a mantis shrimp. Science **303**, 51. (10.1126/science.1089803) 14615546

[34] Ze-Lin W, Ngan-Kee N, Teo SLM, Parra-Velandia FJ. 2012 Fluorescent patterns in some Portunus species (Crustacea: Brachyura: Portunidae). Contrib. Mar. Sci. 135–143.

[35] Poding LH, Jägers P, Senen B, Limmon GV, Herlitze S, Huhn M. 2024 New observations of fluorescent organisms in the Banda sea and in the Red Sea. PLoS One **19**, e0292476. (10.1371/journal.pone.0292476) 38865289 PMC11168664

[36] Bähr S, van der Meij SET. 2019 Red fluorescence in symbiotic coral-dwelling gall crabs. Galaxea J. Coral Reef Stud. **21**, 27–28. (10.3755/galaxea.21.1_27)

[37] Bähr S, Johnson ML, Berumen ML, Hardenstine RS, Rich WA, van der Meij SET. 2021 Morphology and reproduction in the Hapalocarcinus marsupialis Stimpson, 1859 species complex (Decapoda: Brachyura: Cryptochiridae). J. Crustac. Biol. **41**. (10.1093/jcbiol/ruab052)

[38] Wei T *et al*. 2013 Gall polymorphism of coral-inhabiting crabs (Decapoda, Cryptochiridae): a new perspective. J. Mar. Sci. Technol. **21**, J. (10.6119/JMST-013-1223-7)

[39] Fize A, Serène R. 1957 Les Hapalocarcinidés du Viet-Nam. Archives du Museum national d'Histoire naturelle, Paris, Sèptieme Série **5**, i-xiii + 1–202, pls. I–XVIII–202.

[40] Kropp RK. 1990 Revision of the genera of gall crabs (Crustacea: Cryptochiridae) occurring in the Pacific Ocean. Pac. Sci. **44**, 417–448.

[41] Baeza J, Thiel M. 2007 The mating system of symbiotic crustaceans: a conceptual model based on optimality and ecological constraints. In Evolutionary ecology of social and sexual systems (eds J Duffy, M Thiel), pp. 250–269. Oxford, UK: Oxford University Press.

[42] Asakura A. 2009 The evolution of mating systems in decapod crustaceans. In Decapod crustacean phylogenetics (eds J Martin, K Crandall, D Felder), pp. 133–194. Boca Raton, FL: CRC Press.

[43] Rueden C, Schindelin J, Hiner M, DeZonia B, Walter A, Arena E. 2017 ImageJ2: ImageJ for the next generation of scientific image data. BMC Bioinform. **18**, 1–26. (10.1186/s12859-017-1934-z) PMC5708080 29187165

[44] Meadows MG, Anthes N, Dangelmayer S, Alwany MA, Gerlach T, Schulte G, Sprenger D, Theobald J, Michiels NK. 2014 Red fluorescence increases with depth in reef fishes, supporting a visual function, not UV protection. Proc. R. Soc. B **281**, 20141211. (10.1098/rspb.2014.1211) PMC4123709 25030989

[45] Husson F, Josse J, Pages J. 2010 Principal component methods-hierarchical clustering-partitional clustering: why would we need to choose for visualizing data. Appl. Math. Dep. **17**, 1–23.

[46] R Core Team. 2022 R: a language and environment for statistical computing. Vienna, Austria: R Foundation for Statistical Computing. See https://www.R-project.org/.

[47] Warton D, Hui F. 2011 The arcsine is asinine: the analysis of proportions in ecology. Ecology **92**, 3–10. (10.1890/10-0340.1) 21560670

[48] Josse J, Husson F. 2012 Handling missing values in exploratory multivariate data analysis methods. J. Soc. Fr. Stat. **153**, 79–99.

[49] Josse J, Husson F. 2016 missMDA: a package for handling missing values in multivariate data analysis. J. Stat. Softw. **70**, 1–31. (10.18637/jss.v070.i01)

[50] Husson F, Josse J, Le S, Mazet J, Husson MF. 2016 FactoMineR: multivariate exploratory data analysis and data mining. See https://cran.r-project.org/web/packages/FactoMineR/index.html.

[51] Kaiser H. 1960 The application of electronic computers to factor analysis. Educ. Psychol. Meas. **20**, 141–151. (10.1177/001316446002000116)

[52] Wolfe JM, Breinholt JW, Crandall KA, Lemmon AR, Lemmon EM, Timm LE, Siddall ME, Bracken-Grissom HD. 2019 A phylogenomic framework, evolutionary timeline and genomic resources for comparative studies of decapod crustaceans. Proc. R. Soc. B **286**, 20190079. (10.1098/rspb.2019.0079) PMC6501934 31014217

[53] Andrews S. 2010 FastQC: a quality control tool for high throughput sequence data. See https://www.bioinformatics.babraham.ac.uk/projects/fastqc/.

[54] Ewels P, Magnusson M, Lundin S, Käller M. 2016 MultiQC: summarize analysis results for multiple tools and samples in a single report. Bioinformatics **32**, 3047–3048. (10.1093/bioinformatics/btw354) 27312411 PMC5039924

[55] Martin M. 2011 Cutadapt removes adapter sequences from high-throughput sequencing reads. EMBnet J. **17**, 10–12. (10.14806/ej.17.1.200)

[56] Johnson MG, Gardner EM, Liu Y, Medina R, Goffinet B, Shaw AJ, Zerega NJC, Wickett NJ. 2016 HybPiper: extracting coding sequence and introns for phylogenetics from high‐throughput sequencing reads using target enrichment. Appl. Plant Sci. **4**, apps.1600016. (10.3732/apps.1600016) 27437175 PMC4948903

[57] Faircloth B. 2016 PHYLUCE is a software package for the analysis of conserved genomic loci. Bioinformatics **32**, 786–788. (10.1093/bioinformatics/btv646) 26530724

[58] Katoh K, Asimenos G, Toh H. 2009 Multiple alignment of DNA sequences with MAFFT. In Bioinformatics for DNA sequence analysis (ed D Posada), pp. 39–64. Humana Press. (10.1007/978-1-59745-251-9_3) 19378139

[59] Nguyen LT, Schmidt HA, von Haeseler A, Minh BQ. 2015 IQ-TREE: a fast and effective stochastic algorithm for estimating maximum-likelihood phylogenies. Mol. Biol. Evol. **32**, 268–274. (10.1093/molbev/msu300) 25371430 PMC4271533

[60] Kalyaanamoorthy S, Minh BQ, Wong TKF, von Haeseler A, Jermiin LS. 2017 ModelFinder: fast model selection for accurate phylogenetic estimates. Nat. Methods **14**, 587–589. (10.1038/nmeth.4285) 28481363 PMC5453245

[61] Maddison WP, Maddison DR. 2023 Mesquite: a modular system for evolutionary analysis. Version **3**, 81. http://www.mesquiteproject.org

[62] Michels J. 2007 Confocal laser scanning microscopy: using cuticular autofluorescence for high resolution morphological imaging in small crustaceans. J. Microsc. **227**, 1–7. (10.1111/j.1365-2818.2007.01787.x) 17635653

[63] DecaNet. 2024 Cryptochiridae Paulson, 1875. See https://www.decanet.info/aphia.php?p=taxdetails&amp;id=106753.

[64] van der Meij SET. 2015 Origin and diversification of coral-dwelling gall crabs (Cryptochiridae). PhD thesis,Leiden University, The Netherlands.

[65] Xu T, Bravo H, Paulay G, Van der Meij SET. 2022 Diversification and distribution of gall crabs (Brachyura: Cryptochiridae: Opecarcinus) associated with Agariciidae corals. Coral Reefs **41**, 699–709. (10.1007/s00338-021-02163-1)

[66] Stimpson W. 1859 Hapalocarcinus marsupialis, a remarkable new form of brachyurous crustacean on the coral reefs of Hawaii. Proc. Boston Soc. Nat. Hist. **6**, 412–413.

[67] van der Meij SET. 2017 The coral genus Caulastraea Dana, 1846 (Scleractinia, Merulinidae) as a new host for gall crabs (Decapoda, Cryptochiridae), with the description of Lithoscaptus tuerkayi sp. nov.. Crustaceana **90**, 1027–1038. (10.1163/15685403-00003607)

[68] Claassen JR, Tuti Y, van der Meij SET. 2024 An Indo-West Pacific distribution for the coral-dwelling gall crab Lithoscaptus doughnut (Decapoda: Cryptochiridae). Arthropoda **2**, 66–75. (10.3390/arthropoda2010005)

[69] Silva DJA, Erickson MF, dos Santos Guidi R, Pessoa DMA. 2022 Thin-fingered fiddler crabs display a natural preference for UV light cues but show no sensory bias to other hypertrophied claw colouration. Behav. Processes **200**, 104667. (10.1016/j.beproc.2022.104667) 35661795

[70] van der Meij SET, Fransen CHJM, Pasman LR, Hoeksema BW. 2015 Phylogenetic ecology of gall crabs (Cryptochiridae) as associates of mushroom corals (Fungiidae). Ecol. Evol. **5**, 5770–5780. (10.1002/ece3.1808) 26811752 PMC4717343

[71] Cronin TW, Porter ML. 2008 Exceptional variation on a common theme: the evolution of crustacean compound eyes. Evolution **1**, 463–475. (10.1007/s12052-008-0085-0)

[72] Djamgoz M, Vallerga S, Wagner H. 1999 Functional organization of the outer retina in aquatic and terrestrial vertebrates: Comparative aspects and possible significance to the ecology of vision. In Adaptive mechanisms in the ecology of vision (eds S Archer, M Djamgoz, E Loew, J Partridge, S Vallerga), pp. 329–382. Berlin, Germany: Springer. (10.1007/978-94-017-0619-3_11)

[73] Bähr S, van der Meij SET, Terraneo T, Oury N, Michiels NK, Ogg S, Marchese F, Benzoni F. 2025 Data from: Integrative phylogenomics sheds light on the diversity and evolution of fluorescence in coral-dwelling gall crabs. Figshare (10.6084/m9.figshare.27175374)40068824

[74] Bähr S, van de Meij SET, Terraneo T, Oury N, Michiels NK, Ogg S, Marchese F, Benzoni F. 2025 Supplementary material from: Integrative phylogenomics sheds light on the diversity and evolution of fluorescence in coral-dwelling gall crabs. Figshare (10.6084/m9.figshare.c.7702951)40068824

